# Supplementary material for: Two types of axonal muscarinic acetylcholine receptors mediate formation of saliva cocktail in the tick Ixodes ricinus
Source: Nat Commun. 2026 Jan 23;17:2867. doi: 10.1038/s41467-026-68654-3 (PMC13022164; doi:10.1038/s41467-026-68654-3)
Supplement: Supplementary file 1 — Supplementary Information [file 41467_2026_68654_MOESM1_ESM.pdf]

Two Types of Axonal Muscarinic Acetylcholine Receptors Mediate Formation of Saliva Cocktail in the Tick *Ixodes ricinus*

Cáinà Nìng[1], James J. Valdés[2,3], Lourdes Mateos-Hernández[1], Sabine Rakotobe[1], Lianet Abuin-Denis[1,4], Nadia Haddad[1], Lívia Šofranková[1,5], Mirko Slovák[6], Khalid Boussaine[7,] Alison Cartereau[7], Emiliane Taillebois[7], Houssam Attoui[8], Helena Frantová[2], Veronika Urbanová[2], Tereza Kozelková[2,9], Filip Dyčka[9], Petr Kopáček[2], Ondřej Hajdušek[2], Radek Šíma[2,10,11], Jiří Týč[2], Tomáš Bílý[2,9], Martina Tesařová[2], Marie Vancová[2,9], Jan Perner[2], Steeve H. Thany[7,12] and Ladislav Šimo[1,*]

[1]ANSES, INRAE, Ecole Nationale Vétérinaire d'Alfort, UMR BIPAR, Laboratoire de Santé Animale, 94700 Maisons-Alfort, France
[2]Institute of Parasitology, Biology Centre, Czech Academy of Sciences, Branišovská 31, 37005 České Budějovice, Czech Republic
[3]Centre Algatech, Institute of Microbiology, Czech Academy of Sciences, Novohradská 237, 37901 Třeboň, Czech Republic
[4]Animal Biotechnology Department, Center for Genetic Engineering and Biotechnology, Avenue 31 between 158 and 190, P.O. Box 6162, Havana 10600, Cuba
[5]Department of Animal Physiology, Pavol Jozef Šafárik University in Košice, Šrobárova 2, 04180, Košice, Slovak Republic
[6]Institute of Zoology v. v. i, Slovak Academy of Sciences, Dúbravská cesta 9, 84506 Bratislava, Slovakia
[7]University of Orleans, P2E USC-INRAE 1328, 1 rue de Chartres, 45067 Orléans Cedex, France
[8]Ecole Nationale Vétérinaire d'Alfort, Anses, INRAE, Laboratoire de Santé Animale,VIROLOGIE, F-94700, Maisons-Alfort, France
[9]Faculty of Science, University of South Bohemia, České Budějovice, 37005, Czech Republic
[10]Bioptic laboratory, Mikulášské náměstí 4, 32600 Plzeň, Czech Republic
[11]Sikl's Department of Pathology, Faculty of Medicine in Plzen, Charles University, and University Hospital, E. Beneše 13, 305 99 Plzeň, Czech Republic
[12]Institut Universitaire de France (IUF), 1 rue Descartes, 75005 Paris, France

[*]Correspondence: ladislav.simo@vet-alfort.fr

This PDF file includes:

Supplementary Figures 1 to 13
Supplementary Tables 1 and 2
Supplementary References

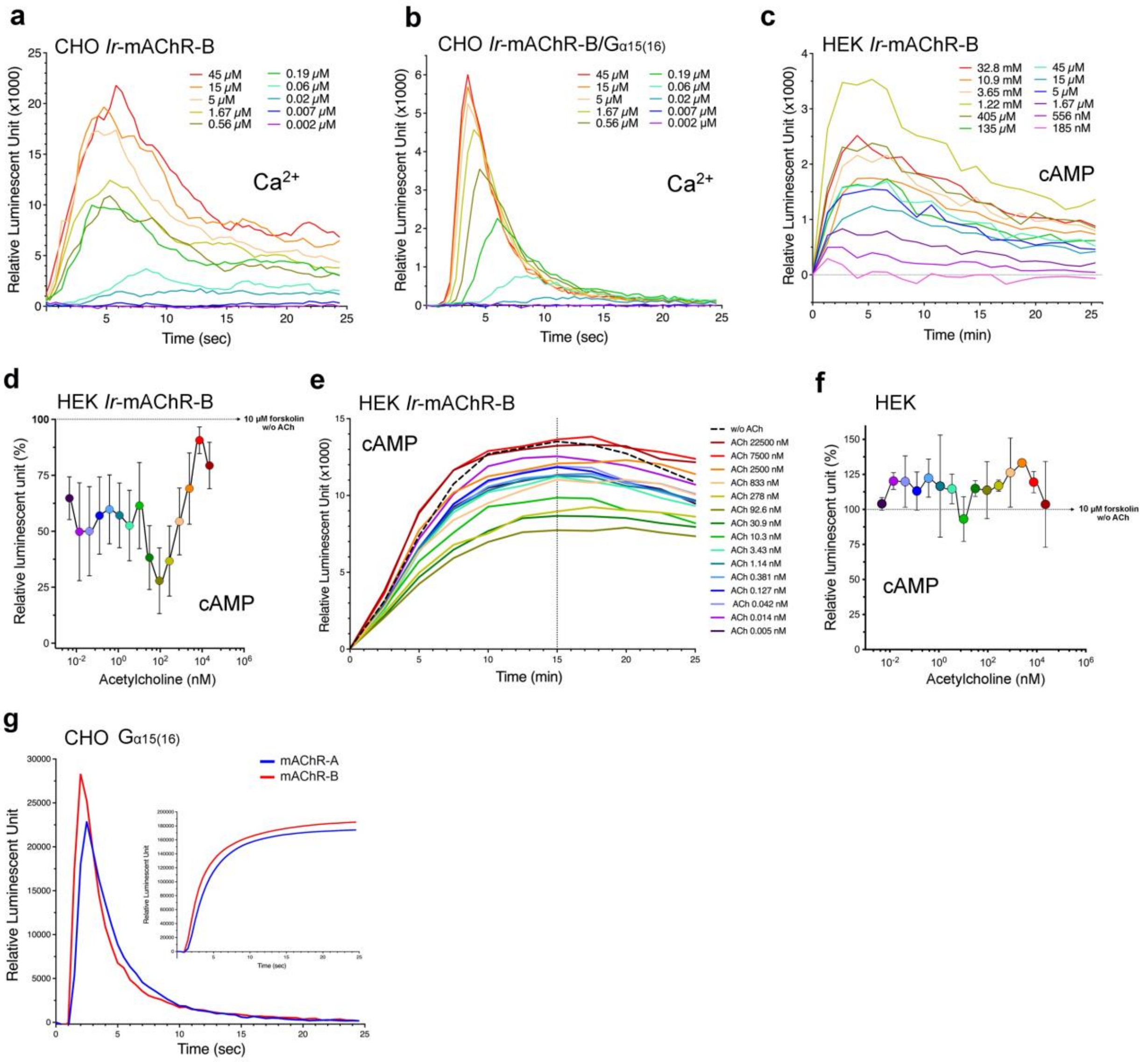

Supplementary Figure 1. Functional responses of *Ir*-mAChR-A and -B to ACh stimulation in heterologous expression systems.

a, b Typical 25 s cellular responses showing ACh-mediated calcium mobilisation via *Ir*-mAChR-B expressed in CHO cells without (**a**) or with (**b**) co-expression of $G_{\alpha15(16)}$. **c** Typical 25 min cellular responses showing ACh-mediated cAMP elevation via *Ir*-mAChR-B expressed in HEK cells. See Fig. 1d–f in the main text. **d** cAMP assay showing responses of *Ir*-mAChR-B-transfected HEK cells pre-treated with a range of ACh concentrations (0.005–22,500 nM, as shown in e) followed by stimulation with 10 μM forskolin. **e** Typical 25 min cellular responses of ACh-pre-treated HEK cells (as in d) subsequently stimulated with 10 μM forskolin. The dotted line indicates the time point used for dose–response analyses. **f** Forskolin responses of mock-transfected HEK cells pre-treated with the same ACh concentrations as in **d**. **g** Typical 25 s cellular responses of *Ir*-mAChR-A and *Ir*-mAChR-B expressed in CHO cells co-expressing $G_{\alpha15(16)}$ and stimulated with 5 μM ACh. Luminescent signals were recorded, with the inset showing cumulative luminescence values. This dataset illustrates the relative responsiveness of each receptor to ACh, which served as a reference for normalising responses to other compounds (also tested at 5 μM) targeting these receptors, as shown in Fig. 1g in the

main text. (d) Data are from three independent transfection replicates (*N* = 3); (f) from two independent transfections (*N* = 2). Statistics: data are mean ± SD. Source data are available in the Source Data file.

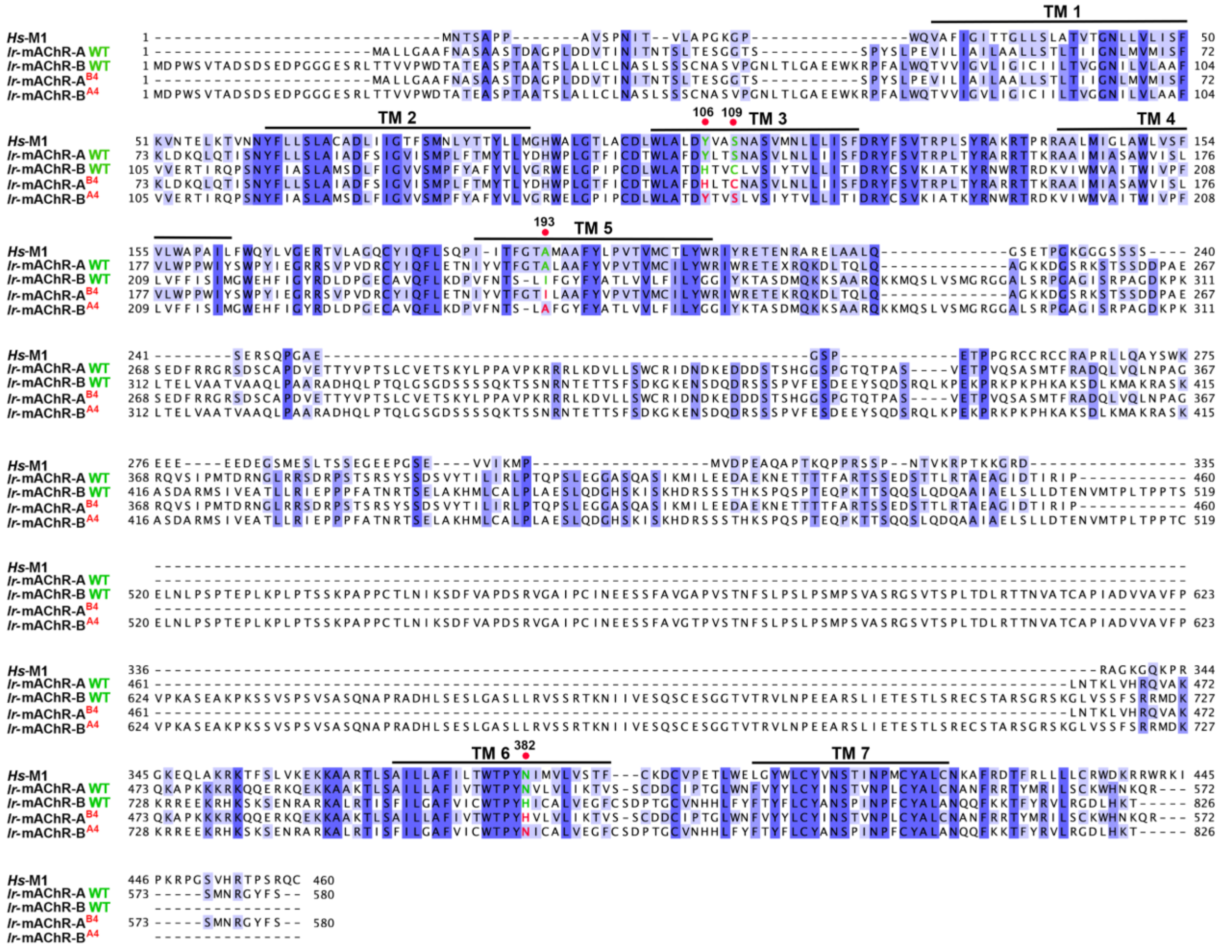

Supplementary Figure 2. Alignment of wild-type and mutant *Ir*-mAChR-A and -B protein sequences with human M1 receptor.

Alignment of translated protein sequences of *Homo sapiens* (Hs) M1 and *I. ricinus* mAChR-A and -B wild-type (WT, green) and mutated variants (mAChR-A^{4B} and -B^{4A}, red). TM1–TM7 denote the predicted transmembrane segments of *Hs*-M1. Red dots indicate amino acid substitution sites, numbered according to the *Hs*-M1 sequence; green and red represent WT and mutated residues, respectively. Shades of Majorelle blue indicate identical amino acids.

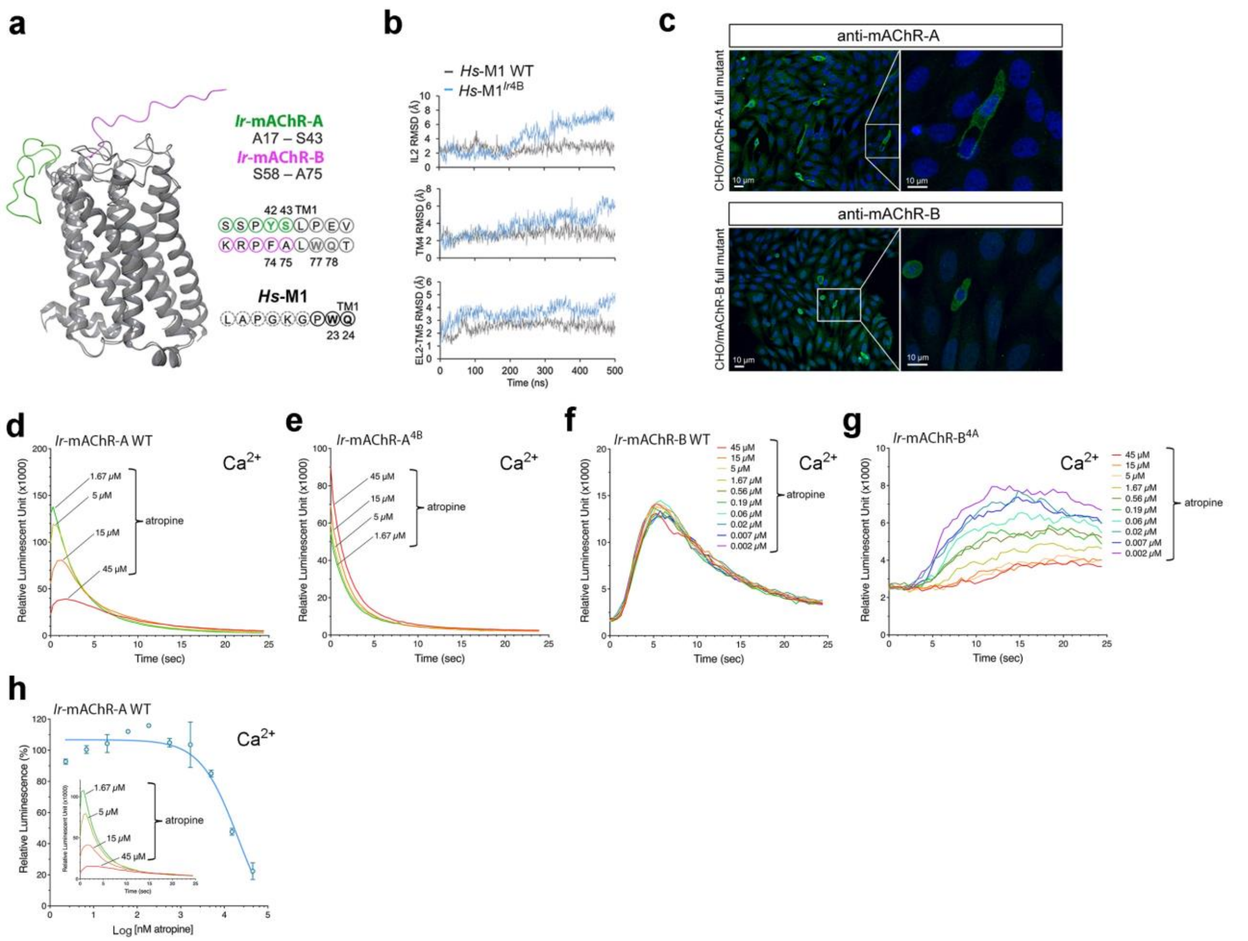

Supplementary Fig. 3 Structural and functional characterization of *Ir*-mAChR-A and -B mutants.

a Structural alignment of the seven *Ir*-mAChR transmembrane domains (TMs, grey) depicting the disordered N-termini of *Ir*-mAChR-A (green) and *Ir*-mAChR-B (magenta). Portions of the mAChR N-termini were truncated for clarity (residue numbers indicated, top right). Circles mark the extracellular *Ir*-mAChR primary sequences (mid-right) forming a conserved membrane-proximal pair (bold and coloured) with mammalian adrenoceptors[1] of the human mAChR (*Hs*-M1, bottom right). Circles terminate at the residue preceding the membrane-embedded region. Dashed circles denote residues absent in the resolved *Hs*-M1 structure[2]. **b** Structural deviations caused by *Hs*-M1 active-site mutations. The 500-ns MD simulations (x-axis) show root mean square deviation (RMSD; y-axis) for wild-type (WT) and full mutant forms at residues 132–134 of IL2 (top), 164–166 of TM4 (middle), and 179–193 of the extracellular loop (EL) 2–TM5 (bottom). **c** Immunodetection of *Ir*-mAChR-A^{4B} and -B^{4A} mutant forms expressed in CHO cells. **d**, **e** Typical 25 s responses showing the effect of varying atropine pre-treatment doses on 33 µM muscarine-mediated responses of *Ir*-mAChR-A WT (d) or *Ir*-mAChR-A^{4B} (e) in CHO cells expressing $G_{\alpha15(16)}$ (see Fig. 2i in the main text). **f**, **g** Typical 25 s responses showing the effect of different atropine pre-treatment doses on 33 µM arecoline-mediated responses of *Ir*-mAChR-B WT (f) or *Ir*-mAChR-B^{4A} (g) in CHO cells expressing $G_{\alpha15(16)}$ (see Fig. 2j in the main text). **h** Dose–response curves representing the inhibitory effect of atropine pre-treatment on 33 µM arecoline-mediated responses of *Ir*-mAChR-A WT in CHO cells expressing $G_{\alpha15(16)}$. Data are from two independent transfection replicates (*N*

= 2). Inset shows typical 25 s responses. This experiment validated atropine's inhibitory effect on arecoline-mediated activation—the most potent agonist for the *Ir*-mAChR-B mutant (see Fig. 2g in the main text)—in the *Ir*-mAChR-A WT context, testing the same agonist–antagonist pair on *Ir*-mAChR-B WT and *Ir*-mAChR-B^{4A} (see Fig. 2j in the main text and panels f, g in current figure). Statistics: (h) nonlinear regression analyses and data are mean ± SD. Source data are available in the Source Data file.

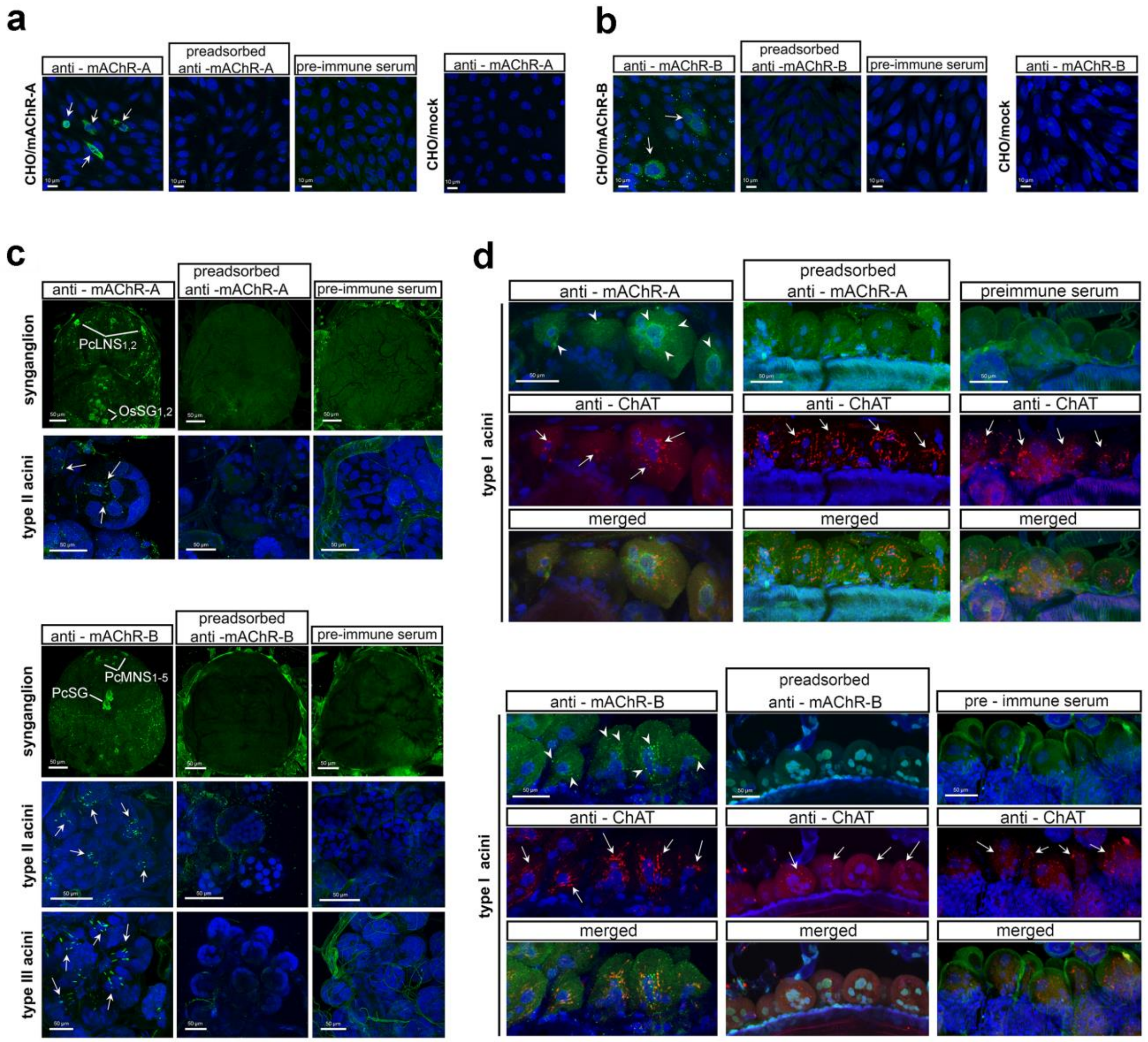

Supplementary Fig. 4. Validation of anti-*Ir*-mAChR-A and anti-*Ir*-mAChR-B antibodies.

a, b Immunoreactivity of CHO cells transfected with either mAChR-A (c) or mAChR-B (d). In each panel, cells were labelled with: (i) the corresponding primary antibody (anti-mAChR-A or -B; left panels, green; arrows), (ii) the antigen-pre-adsorbed antibody (middle panels; no immunoreactivity), and (iii) the corresponding pre-immune serum (right panels; no immunoreactivity). Mock-transfected CHO cells stained with either anti-

mAChR-A or anti-mAChR-B showed no signal. These results arise from two (n = 2) independent experiments and confirm the specificity of both antibodies. Note that both anti-mAChR-A and -B antibodies also recognised the corresponding mutant receptor forms in CHO cells (Supplementary Fig. 3c). Nuclei are counterstained with DAPI (blue). **c** Immunolabelling of unfed female synganglia and salivary gland (SG) acini types II and III using anti-*Ir*-mAChR-A (upper panel) or anti-*Ir*-mAChR-B (lower panel) antibodies. Left columns show positive immunostaining (green; arrows). Middle columns show staining with antigen-pre-adsorbed antibody. Right columns show staining with the corresponding pre-immune serum. Note that both pre-adsorption of the antibodies with antigen and substitution with pre-immune serum abolished *Ir*-mAChR-A and -B staining entirely. (N = 5 ticks). **d** Double immunolabelling of unfed *I. ricinus* female type I SG acini with anti-ChAT (red, arrows) and either anti-mAChR-A (upper panel, green, arrowheads) or anti-*Ir*-mAChR-B (lower panel, green, arrowheads). The left columns show positive immunostaining revealing ChAT-positive axons (red, arrows) and nearby *Ir*-mAChR-A or -B signals (green, arrowheads). Middle columns show staining with antigen-pre-adsorbed antibodies. Right columns show staining with the corresponding pre-immune serum. Note that both pre-adsorption with antigen and substitution with pre-immune serum abolished *Ir*-mAChR-A or -B staining entirely (n = 10 ticks). The specificity of anti-ChAT staining in neurons innervating *I. ricinus* SG type I acini was confirmed by in situ hybridisation in Mateos-Hernández *et al.* (2020)[3]. This double-labelling experiment was conducted under the same conditions as in Fig. 5f of the main text to ensure consistency.

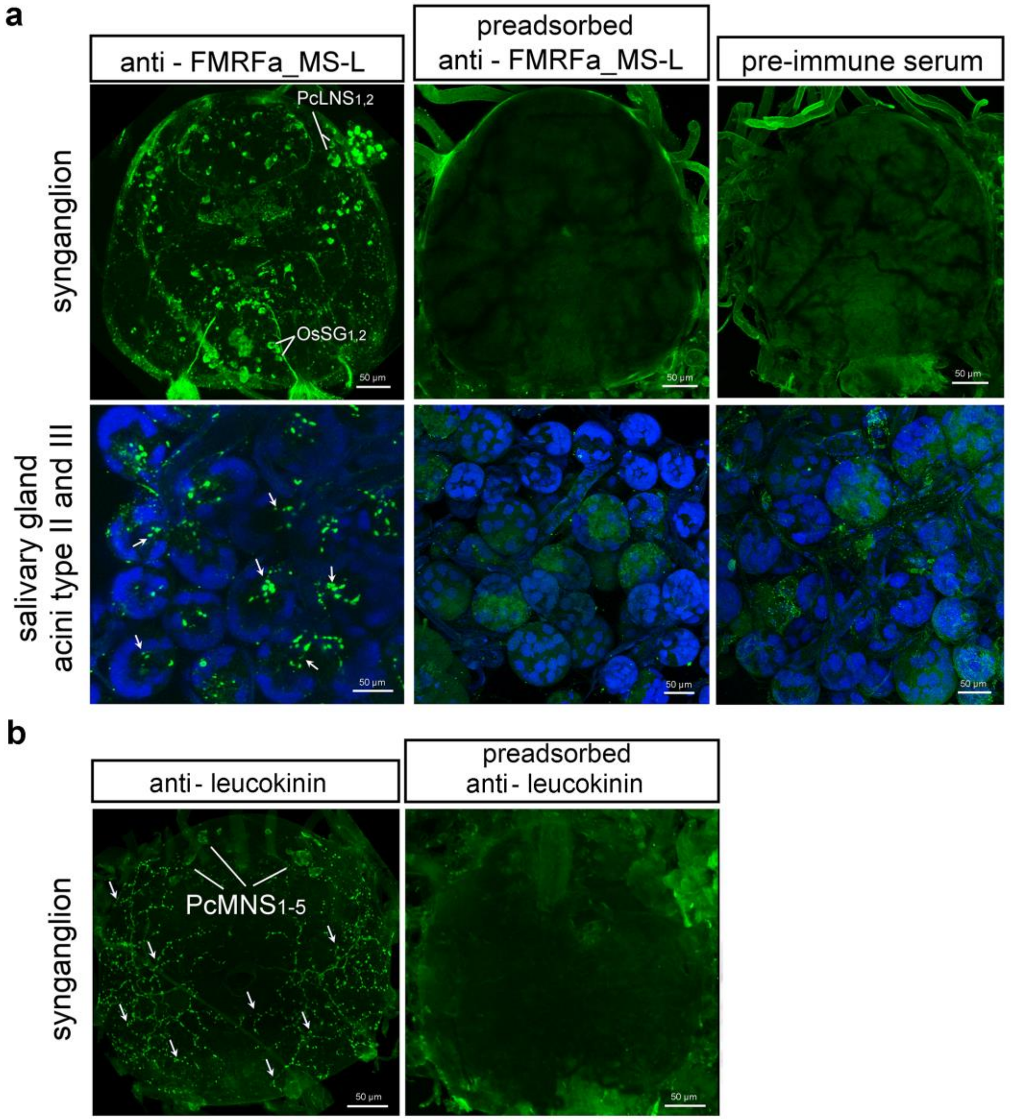

Supplementary Fig. 5. Validation of anti-FMRFa_MS-L and anti-leucokinin antibodies.

a Immunolabelling of unfed female synganglia (upper panel) and salivary glands (SG; lower panel). Left columns show positive immunostaining (green; arrows). Among multiple neurons stained in the positive control (left panel), only $PcLNS_{1,2}$ cells and SG-innervating neurons $OsSG_{1,2}$ are labelled. Middle columns show staining with the antigen-pre-adsorbed antibody. Right columns show staining with the corresponding pre-immune serum. Note that both pre-adsorption of the antibodies with antigen and substitution with pre-immune serum abolished FMRFa_MS-L staining entirely. Anti-FMRFa_MS-L immunoreactivity in SG axons can also be abolished by pre-adsorption of the antibody with SIFamide neuropeptide (see Supplementary Fig. 8b, c), indicating that this antibody binds to SIFamide within the axonal projections reaching the SG (*n* = 5 ticks) **b** Immunolabelling with anti-leucokinin in the unfed female synganglion. The left panel shows positive immunostaining where $PcLNS_{1,2}$ cells are labelled, and arrows indicate the dorso-lateral surface axons (green) of these cells. The right column shows staining with the antigen-pre-adsorbed antibody. Note that pre-adsorption of the antibody with antigen abolished leucokinin staining entirely (*n* = 5 ticks).

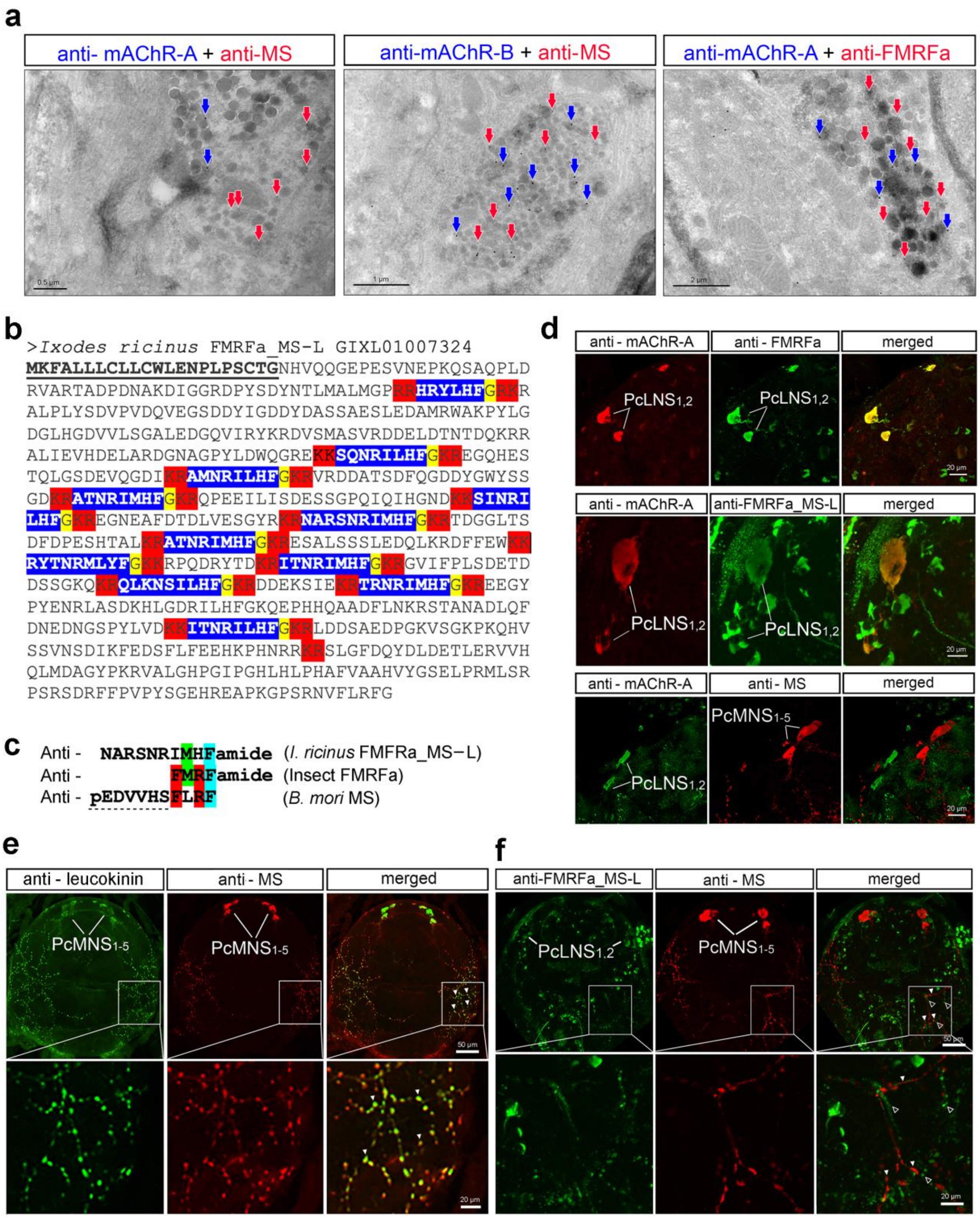
a
anti- mAChR-A + anti-MS
anti-mAChR-B + anti-MS
anti-mAChR-A + anti-FMRFa
0.5 µm
1 µm
2 µm
b
>Ixodes ricinus FMRFa_MS-L GIXL01007324
MKFALLLCLLCWLENPLPSCTGNHVQQGEPESVNEPKQSAQPLD
RVARTADPDNAKDIGGRDPYSDYNTLMALMGPRRHRYLHFGRKR
ALPLYSDVPVDQVEGSDDYIGDDYDASSAESLEDAMRWAKPYLG
DGLHGDVVLSGALEDGQVIRYKRDVSMASVRDDELDTNTDQKRR
ALIEVHDELARDGNAGPYLDWQGREKKSQNRILHFGKREGQHES
TQLGSDEVQGDIKRAMNRILHFGKRVRDDATSDFQGDDYGWYSS
GDKRATNRIMHFGKRQPEEILISDESSGPQIQIHGNDKKSINRI
LHFGKREGNEAFDTDLVESGYRKRNARSNRIMHFGKRTDGGLTS
DFDPESHTALKRATNRIMHFGKRESALSSSLEDQLKRDFFEWKK
RYTNRMLYFGKKRPQDRYTDKRITNRIMHFGKRGVIFPLSDETD
DSSGKQKRQLKNSILHFGKRDDEKSIEKRTRNRIMHFGKREEGY
PYENRLASDKHLGDRILHFGKQEPHHQAADFLNKRSTANADLQF
DNEDNGSPYLVDKKITNRILHFGKRLDDSAEDPGKVSGKPKQHV
SSVNSDIKFEDSFLFEEHKPHNRRKRSLGFDQYDLDETLERVVH
QLMDAGYPKRVALGHPGIPGHLHLPHAFVAAHVYGSELPRMLSR
PSRSDRFFPVPYSGEHREAPKGPSRNVFLRFG
c
Anti - NARSNRIMHFamide (I. ricinus FMFRa_MS−L)
Anti - FMRFamide (Insect FMRFa)
Anti - pEDVVHSFLRF (B. mori MS)
d
anti - mAChR-A
anti - FMRFa
merged
PcLNS1,2
20 µm
anti - mAChR-A
anti-FMRFa_MS-L
merged
PcLNS1,2
20 µm
anti - mAChR-A
anti - MS
merged
PcMNS1-5
PcLNS1,2
20 µm
e
anti - leucokinin
anti - MS
merged
PcMNS1-5
50 µm
20 µm
f
anti-FMRFa_MS-L
anti - MS
merged
PcLNS1,2
PcMNS1-5
50 µm
20 µm

Supplementary Figure 6. Double labelling with anti-*Ir*-mAChR-A or -B and neuropeptide antibodies in *I. ricinus* unfed female synganglion.

a TEM micrographs showing double immunogold labelling in axons located in the dorso-lateral perineurium of the synganglion. Images represent enlarged, uncoloured insets from Fig. 3o in the main text. Blue and red arrows indicate 15 nm and 10 nm particles, respectively. Colour-coded arrows point to the sites of immunoreaction (*n* = 4 ticks). **b** Predicted structure of the *I. ricinus* FMRFa_myosuppressin-like (FMRFa_MS-L) prepropeptide. The signal peptide is shown in bold underlined text, predicted peptides are white letters on a blue background, processing (cleavage) sites are shaded in red, and putative glycine-derived C-terminal amidation sites are highlighted in yellow. **c** Alignment of *I. ricinus* FMRFa_MS-L (against which an antibody was generated in this study), insect FMRFa, and *B. mori* MS mature neuropeptides (see Supplementary Table 2). Coloured backgrounds indicate identical amino acid residues shared at the C-terminus. Note that the antibody against *B. mori* MS recognises the N-terminus of the peptide (underlined dotted line)[4]. **d** Detail of lateral neurosecretory cell (NSC) bodies double labelled with anti-mAChR-A and neuropeptide antibodies raised either against FMRFa, FMRFa_MS-L or MS (see panel c). Note that antibodies against *Ir*-mAChR-A and FMRFa_MS-L, as well as the insect FMRFa antibody, recognised the same lateral $PcLNS_{1,2}$ cells (yellow), whereas the antibody raised against *B. mori* MS recognised medial $PcMNS_{1–5}$ cells (Figs. 3n and 4a in the main text) (n= 4-5 ticks stained in ≥4 independent experiments). **e** Colocalization (yellow in merged images) of anti-leucokinin (green) and anti-MS (red) antibodies in medial $PcMNS_{1–5}$ cells and their axons (arrowheads) at the dorso-lateral surface region of the synganglion. **f** Double labelling with anti-FMRFa_MS-L (green) and anti-MS (red) antibodies in the synganglion. Note that each antibody stained distinct sets of NSCs: $PcLNS_{1,2}$ or $PcMNS_{1–5}$, respectively, including their axons (open and filled arrowheads, respectively) at the dorso-lateral synganglion surface (*n* = 5 ticks). See the summarising schema in Fig. 4d of the main text.

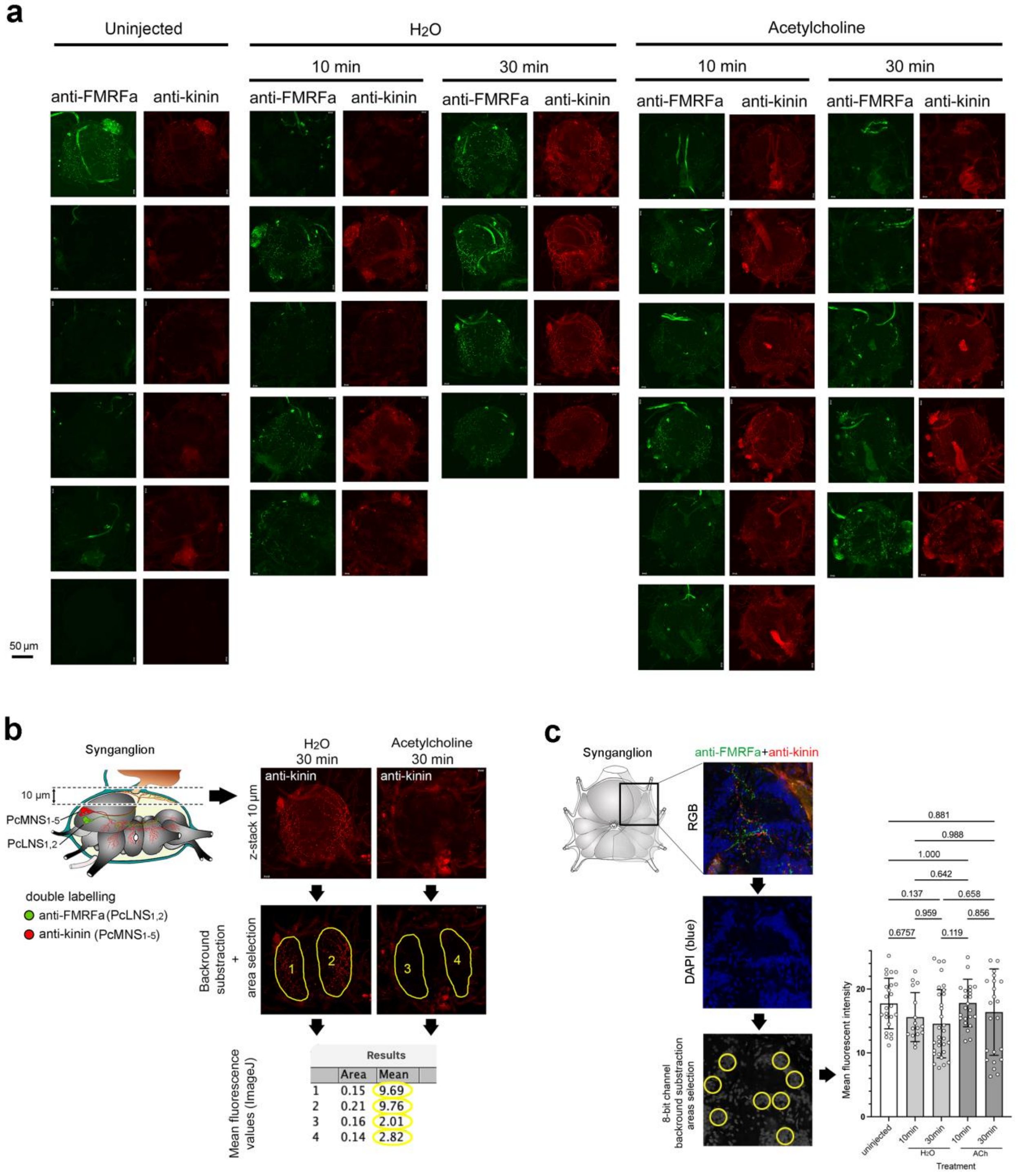

Supplementary Figure 7. Quantification of fluorescent signal in dorso-lateral axonal arborisations originating either from PcLNS$_{1,2}$ or PcMNS$_{1–5}$ cells in uninjected, water-, and ACh-injected *I. ricinus* unfed females, as shown in Fig. 4g of the main text.

a Fluorescent images of all untreated and treated synganglia used for analysis. Time points at 10 min and 30 min post water or ACh injection were examined. All synganglia were simultaneously double-stained with anti-FMRFa (green) and anti-leucokinin (red) antibodies, and images were captured using identical gain and laser sensitivity settings on confocal microscopy (n = 6, 5, 4, 6 and 5 as showed in the figure). **b** Methodological

workflow for fluorescent signal measurement in merged 10 μm z-stack images of the dorso-lateral synganglion. Note that anti-FMRFa was used instead of anti-FMRFa_MS-L in this experiment due to its exclusive recognition of $PcLNS_{1,2}$ dorso-lateral axons. In contrast, the anti-FMRFa_MS-L antibody also labels surface-adjacent neurons in this area (Fig. 4a in the main text and Supplementary Fig. 6d, f), which would confound accurate signal quantification at the dorso-lateral surface of the synganglion. **c** Methodological workflow of DAPI staining measurement, used as a negative control among treated synganglia. DAPI intensities: N = 4, 2, 4, 4, 3 synganglia; n = 24, 16, 31, 25, 25 areas measured across the corresponding synganglia. Multiple areas were measured because DAPI intensity varied slightly across different regions. p - values are shown above the bars. (c) One-way ANOVA (two-sided) followed by Tukey's multiple-comparison post hoc test. Data are mean ± SD. Source data are provided in the Source Data file.

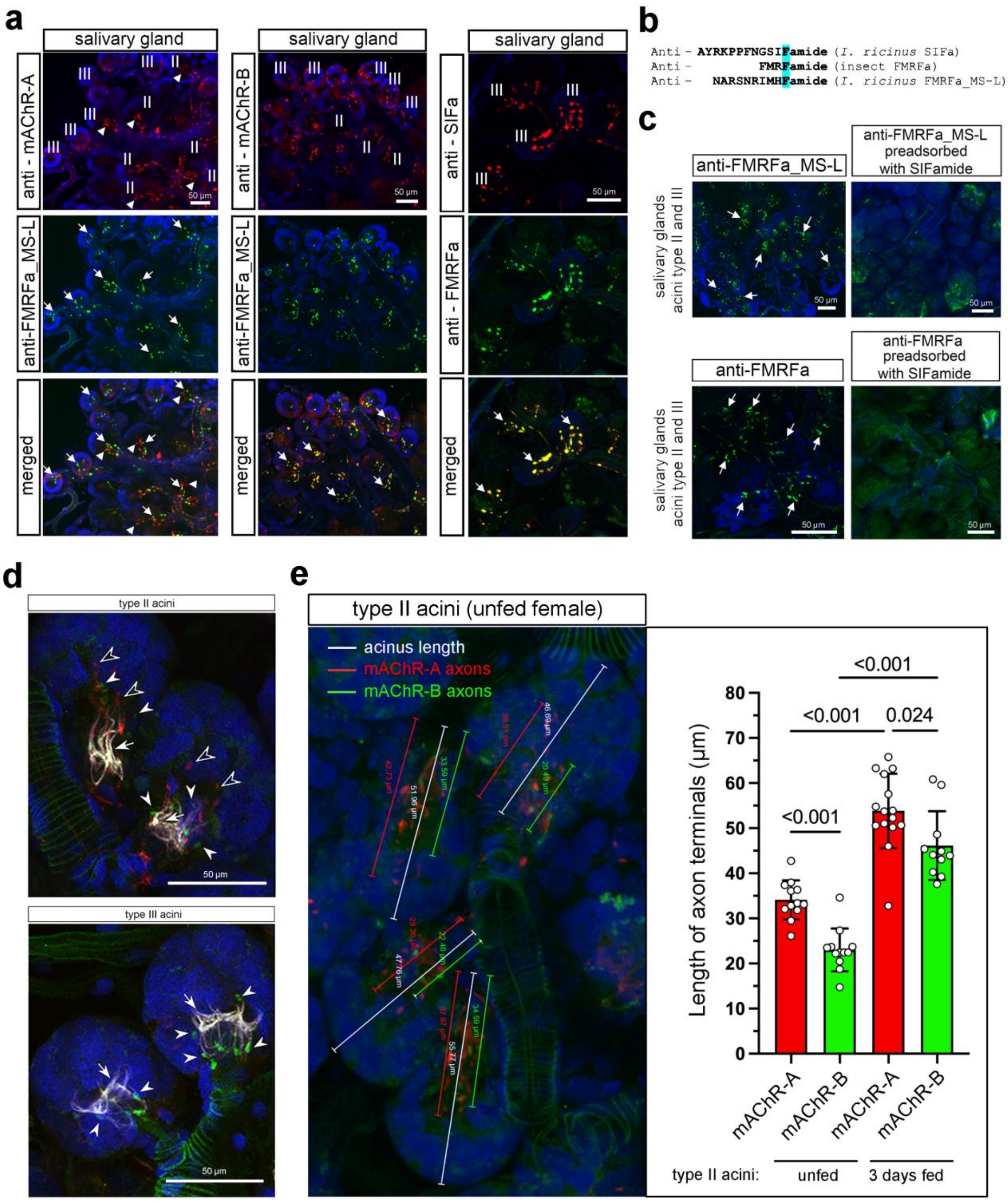

Supplementary Figure 8. Colocalization and specificity of anti-FMRFa_MS-L, anti-FMRFa, anti-SIFa, and anti-*Ir*-mAChR antibodies in *I. ricinus* salivary glands and synganglion.

a Double staining of either anti-*Ir*-mAChR-A or -B with anti-FMRFa_MS-L in axonal projections reaching type II and III acini in an unfed *I. ricinus* female. Left columns: anti-FMRFa_MS-L recognises axons (arrows) innervating both type II and III acini, while anti-*Ir*-mAChR-A (arrowheads) labels axons exclusively in type II acini. Middle columns: colocalization (yellow) of anti-mAChR-B and anti-FMRFa_MS-L is observed in axons

innervating both type II and III acini. Roman numerals indicate the acinus types (see also Figs. 5a, b in the main text). Right columns: colocalization (yellow, arrows) of anti-SIFa and anti-FMRFa in axonal projections reaching both type II and III acini in an unfed *I. ricinus* female (*n* = 4 ticks per each staining). Note that a typical FMRFamide peptide sequence has not been identified in ticks; thus, the anti-FMRFa antibody likely cross-reacts with the SIFamide peptide, similar to the observed cross-reactivity of anti-FMRFa_MS-L. **b** Alignment of *I. ricinus* SIFamide, insect FMRFa, and *I. ricinus* FMRFa_MS-L mature neuropeptides against which antibodies were raised (see Supplementary Table 2). The aqua-blue background indicates a conserved phenylalanine residue at the C-terminus. **c** Pre-adsorption of anti-FMRFa_MS-L with the SIFamide peptide antigen completely abolished immunoreactivity in SG innervation (*n* = 4 ticks). The expression of SIFamide in PsSG neurons and their axons projecting to the tick SG was well characterised by proteomic and in situ hybridisation approaches (Šimo *et al.*, 2009)[5] and further confirmed in the current study (Fig. 5b in the main text). Our results suggest that both anti-FMRFa_MS-L and anti-FMRFa antibodies cross-react with the SIFamide peptide in axonal projections targeting type II and III SG acini. However, to confirm this conclusion, further investigation into the various classes of neuropeptides present in these peptidergic axon terminals and their physiological roles is required. **d** Association of *Ir*-mAChR-A (red; open arrowheads) and *Ir*-mAChR-B (green; filled arrowheads) immunoreactive axons with the myoepithelial cell, visualised by anti-β-tubulin (white, arrows), *n* = 6 ticks in two independent staining experiments). For ultrastructural details of the association between these axons—*Ir*-mAChR-A detected by anti-PDF and *Ir*-mAChR-B by anti-SIFa—and the myoepithelial cell in tick SG, see Vancová *et al.* (2019)[6]. **e** Representative image showing measurement of axon terminal length within type II acini in a double-labelled specimen from an unfed *I. ricinus* female stained with anti-*Ir*-mAChR-A and anti-*Ir*-mAChR-B. Measurement scales were aligned at the base of the acinus. The right panel shows a statistical comparison of the length of axonal terminals within type II acini between unfed and three-day-fed *I. ricinus* females. *N* = 6 and 4 SGs from unfed and three-day-fed females, respectively; *n* = 12 individual type II acini were analysed for the length of mAChR-A and mAChR-B axons. In the three-day-fed SGs, *n* = 15 and 11 type II acini were measured for *Ir*-mAChR-A and *Ir*-mAChR-B axons, respectively. Statistics: (e) one-way ANOVA (two-sided) followed by Tukey's multiple-comparison post hoc test. *p* - values are given above the bars. Data are mean ± SD. Source data are provided in the Source Data file.

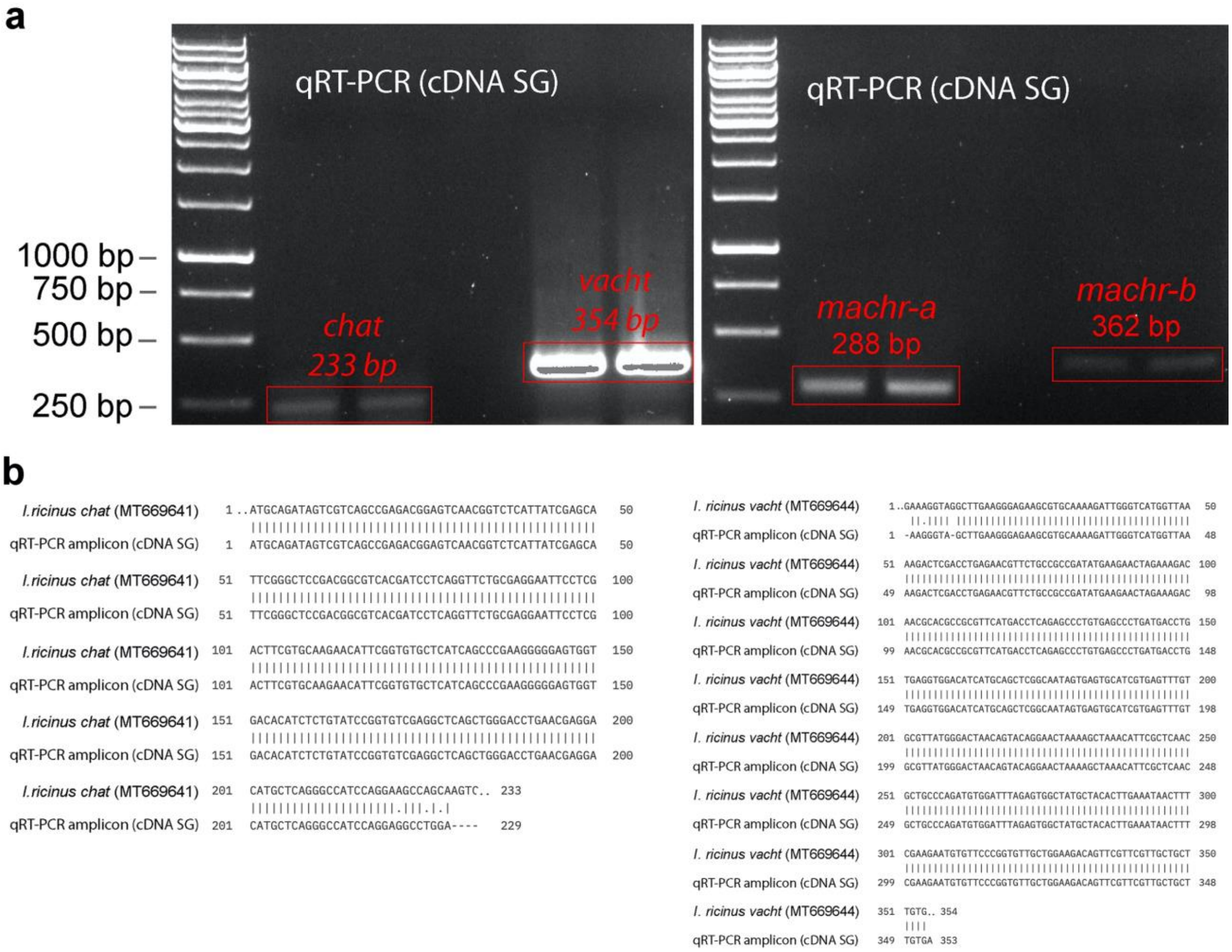

Supplementary Fig. 9. Validation of *I. ricinus* cholinergic gene qRT–PCR amplicons.

a Full-length agarose gel images showing the migration of qRT–PCR products of *chat*, *vacht*, *machr-a*, and *machr-b* amplified from cDNA prepared from partially fed *I. ricinus* female salivary glands. **b** Alignment of *I. ricinus chat* and *vacht* sequences with their corresponding qRT–PCR amplicons from panel a. For primer sequences, see Mateos-Hernández *et al.* (2020)[3].

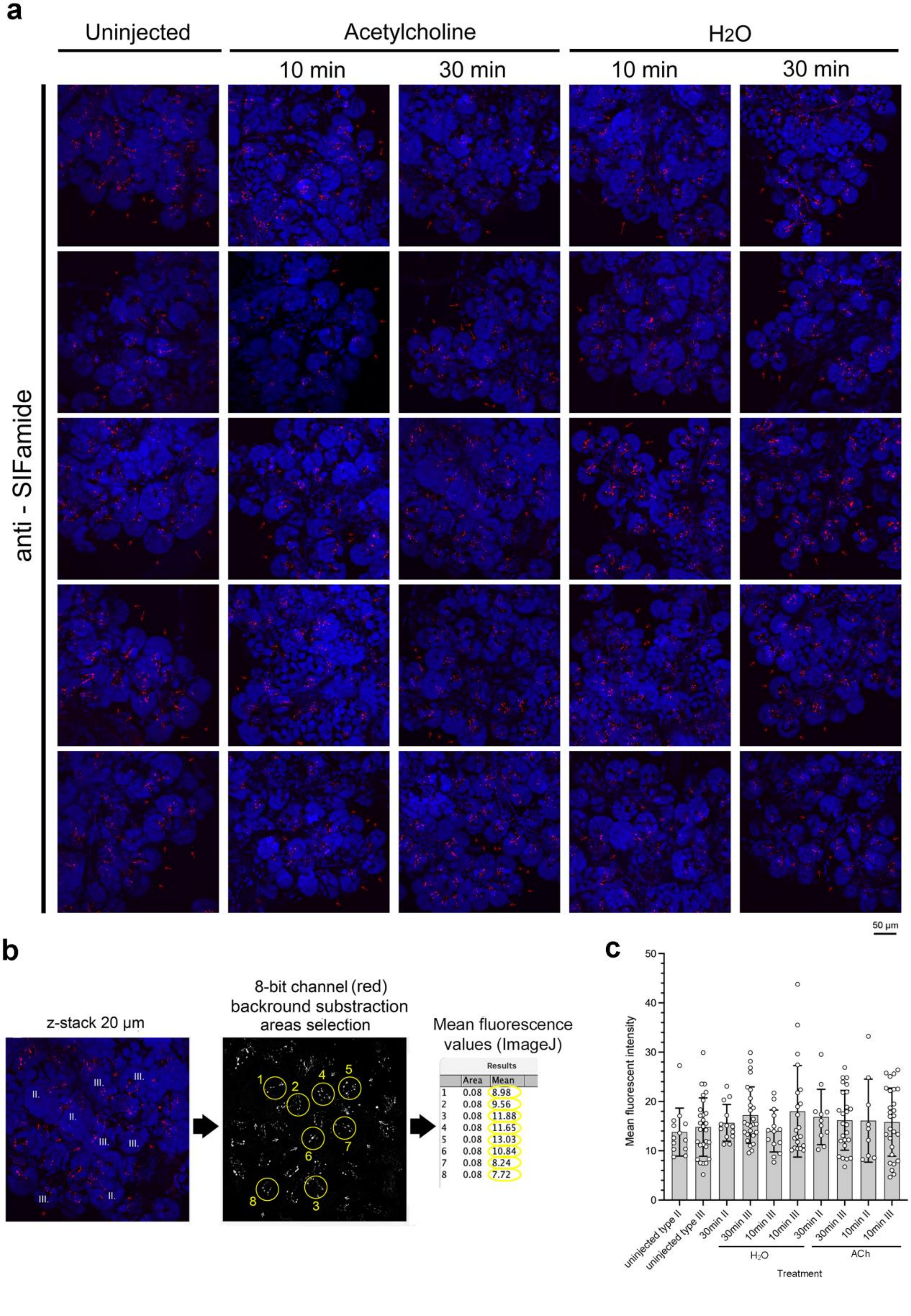
a
Uninjected
Acetylcholine
H2O
10 min
30 min
10 min
30 min
anti - SIFamide
50 µm
b
z-stack 20 µm
8-bit channel (red)
backround substraction
areas selection
Mean fluorescence
values (ImageJ)
Results
Area Mean
1 0.08 8.98
2 0.08 9.56
3 0.08 11.88
4 0.08 11.65
5 0.08 13.03
6 0.08 10.84
7 0.08 8.24
8 0.08 7.72
c
Mean fluorescent intensity
0
10
20
30
40
50
uninjected type II
uninjected type III
30min II
30min III
10min III
10min III
30min II
30min III
10min II
10min III
H2O
ACh
Treatment

Supplementary Figure 10. Quantification of fluorescent signal in axon terminals within type II and III salivary gland acini in uninjected, water-injected, and ACh-injected *I. ricinus* unfed females.

a All untreated and treated salivary glands (SG) were investigated. Time points at 10 min and 30 min post water or ACh injection were examined. All SGs were simultaneously stained with anti-SIFa (red), and fluorescent images were captured under identical gain and laser sensitivity settings using confocal microscopy. **b** Methodological workflow for fluorescent signal quantification from merged z-stack images of axons in SG acini. **c** Statistical comparison of relative fluorescence intensities. *N* = 4 SG per condition as in the figure, with individual acini measured across SG as follows: for uninjected SGs, *n* = 13 type II and 28 type III acini; for H_2O-injected (30 min), *n* = 13 type II and 26 type III acini; for H_2O-injected (10 min), *n* = 14 type II and 21 type III acini; for ACh-injected (30 min), *n* = 11 type II and 25 type III acini; and for ACh-injected (10 min), *n* = 9 type II and 27 type III acini. Statistics: (c) one-way ANOVA (two-sided) followed by Tukey's multiple-comparison post hoc test. No statistically significant differences were detected ($p \geq 0.05$). Data are mean ± SD. Source data are provided in the Source Data file.

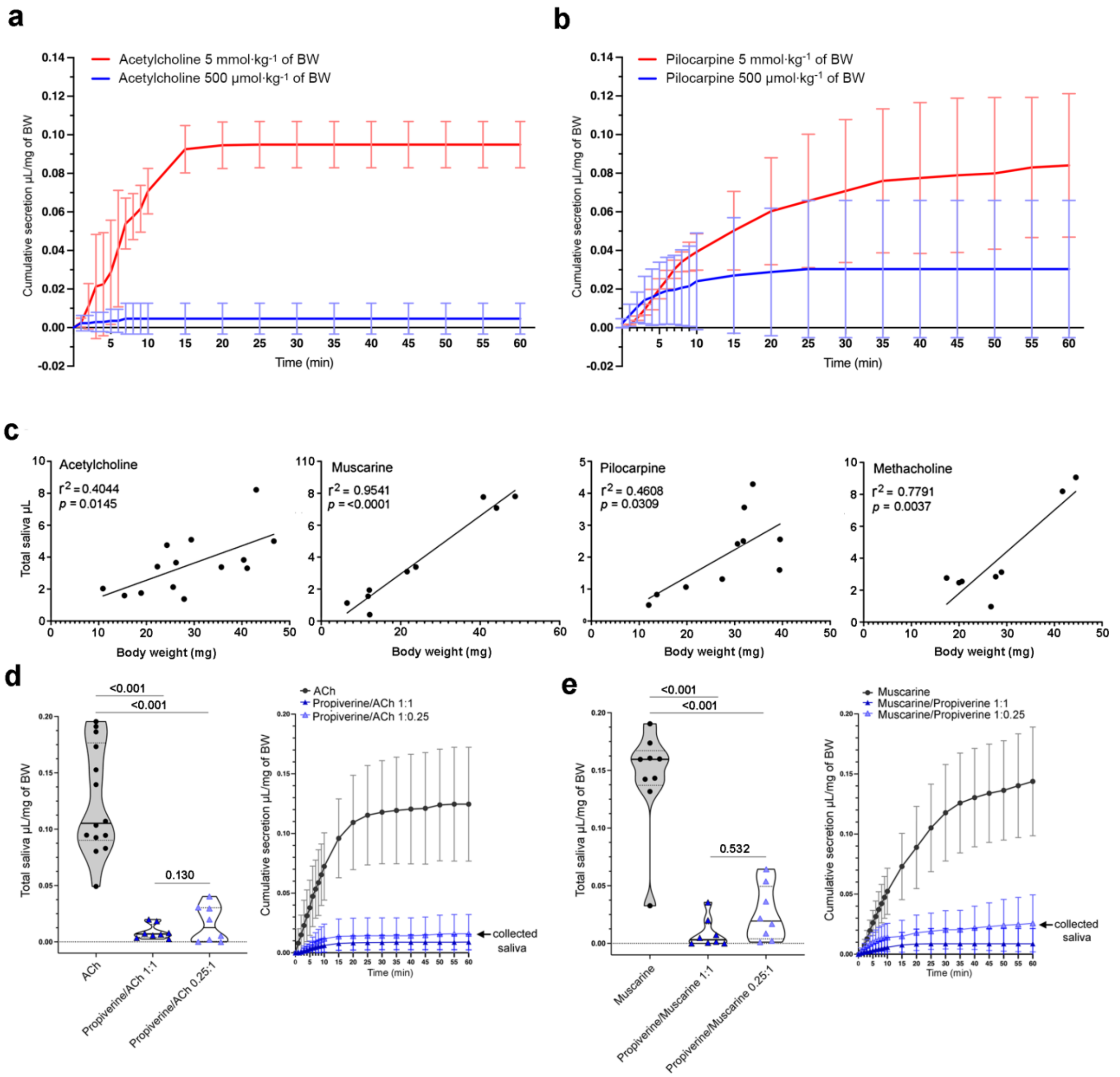

Supplementary Figure 11. Effect of cholinergic agonists and antagonists on salivation in *I. ricinus* females.

a, b Effect of in vivo injection of two different doses of ACh (a) and pilocarpine (b) on the salivation rate of *I. ricinus* females. Blue indicates the effect of 500 µmol/kg body weight (BW), as described in Kaufman (1978)[7]. Red indicates the effect of a tenfold higher concentration, 5 mmol/kg BW, used in the present study. (a, b) *n* = 3 individual female ticks analysed. **c** Correlation between partially fed *I. ricinus* female body weights (BW) and the total volume of saliva secreted within 60 min after injection with each of four different cholinergic agonists. Correlation analyses were performed based on the datasets presented in Figs. 7b–e of the main text. *n* = 14, 9, 10, 8 individual female ticks analysed. **d, e** Comparison of salivation rates triggered by the common agonists ACh (d) or muscarine (e) in females pre-treated with the common antagonist propiverine, followed by agonist injection. This corresponds to experimental conditions 7 and 8 shown in Fig. 7k of the main text. Propiverine: agonist ratios of 1:1 or 0.25:1 were tested. The rationale for this experiment was to obtain saliva for proteomic analyses under conditions where

the activity of both mAChR-A and -B was altered by propiverine. As a 1:1 propiverine:agonist ratio nearly completely inhibited salivation (see Figs. 7b, c, g, h in the main text), a 0.25:1 ratio was applied to allow limited saliva secretion. Left panels in d and e show truncated violin plots comparing total saliva volume secreted over 60 min; right panels show cumulative salivary secretion rates of *I. ricinus* females over 60 min post injection. In the left panels (d, e), n values correspond to the number of individual female ticks analysed: (d) n = 14, 8, 8; (e) n = 9, 8, 8. The same data are summarised as plots in the right panels. Statistics: (d, e, left panels) one-way ANOVA (two-sided) followed by Tukey's multiple-comparison post hoc test; data are presented as median (solid line) with interquartile range (dotted line). p - values are given above the bars. (c) Linear regression analysis and correlation significance analysis. (a, b, d, e) Data are mean ± SD. Source data are provided in the Source Data file.

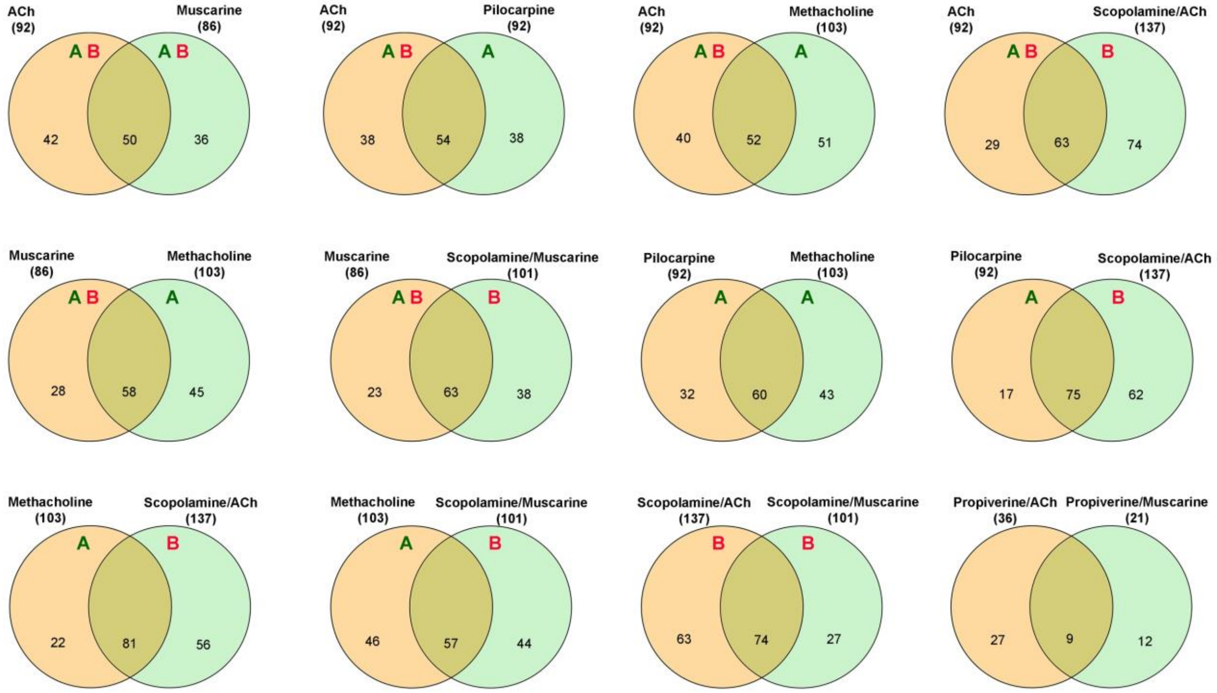

Supplementary Fig. 12. Venn diagrams showing the number of proteins detected in *I. ricinus* saliva following in vivo injection of different chemical agents. Bold red (A) and bold green (B) indicate the mAChR types activated under specific experimental conditions. Analyses of saliva were performed in three technical replicates (n = 3); see Fig. 7n in the main text and Supplementary Dataset 2). The final Venn diagram does not indicate either receptor, as propiverine acts as an antagonist for both mAChR-A and mAChR-B. For this experiment, propiverine was diluted fourfold to obtain saliva while both receptors were simultaneously affected (see Supplementary Fig. 11d, e). This approach enabled saliva collection despite partial antagonism of both receptors by the compound. Source data are provided in the Source Data file.

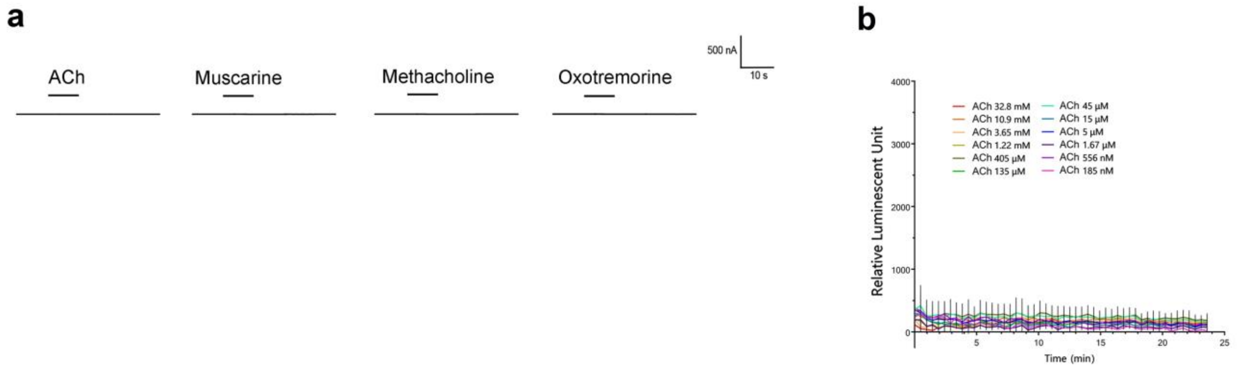

Supplementary Figure 13. Control electrophysiological and luminescence assays in non-transfected systems.

a Electrophysiological recordings following the application of 0.5 µM ACh, muscarine, methacholine, and oxotremorine on non-injected *Xenopus* oocytes. No currents were observed at the tested concentrations. A total of 11 batches (*N* = 11) were tested under control conditions. **b** Mock-transfected HEK cells expressing GloSensor™ showed no responses to various doses of ACh. Note that mock-transfected CHO cells tested with ACh were assessed in our previous study, Mateos-Hernández *et al.* (2020)[3]. Source data are provided in the Source Data file.

Supplementary Table 1. List of chemicals

4-DAMP	Sigma	Cat# SML0255
Aceclidine hydrochloride	Sigma	Cat# SML0180
Acetylcholine chloride	Sigma	Cat# A6625
Arecoline hydrobromide	Sigma	Cat# 31593
Atropine Sulfate Crystalline	Sigma	Cat# A0257
Benztropine mesylate	Sigma	Cat# SML0847
Bethanechol chloride	Sigma	Cat# C5259
BQCA	Sigma	Cat# SML0497
Carbamoylcholine chloride	Sigma	Cat# C4382
Cevimeline hydrochloride hemihydrate	Sigma	Cat# SML0007
Chlorpromazine hydrochloride	Sigma	Cat# 31679
Dicyclomine hydrochloride	Sigma	Cat# D7909
Dimenhydrinate	Sigma	Cat# D2396
Diphenhydramine hydrochloride	Sigma	Cat# D3630
Gallamine triethiodide	Sigma	Cat# G8134
Glycopyrrolate	Sigma	Cat# SML0029
Haloperidol solution	Sigma	Cat# H-030
Hydralazine hydrochloride	Sigma	Cat# H1753
Hydroxyzine dihydrochloride	Sigma	Cat# H8885
Imipramine hydrochloride	Sigma	Cat# I7379
McN-A-343	Sigma	Cat# C7041
Methacholine chloride	Sigma	Cat# A2251
Metoclopramide hydrochloride	Sigma	Cat# M0763
(+)-Muscarine chloride	Sigma	Cat# M6532
N-Desmethylclozapine	Sigma	Cat# D5676
Olanzapine	Sigma	Cat# O1141
Oxotremorine M	Sigma	Cat# O100
Pilocarpine hydrochloride	Sigma	Cat# P6503
Propantheline bromide	Sigma	Cat# P8891
Propiverine hydrochloride	Sigma	Cat# SML0602
(-)-Scopolamine hydrobromide trihydrate	Sigma	Cat# S1875
Solifenacin Succinate	Sigma	Cat# SML2141
Telenzepine dihydrochloride hydrate	Sigma	Cat# T122
tert-Butyl peroxybenzoate	Sigma	Cat# 77200
Tolterodine L-tartrate	Sigma	Cat# SML3034
Tropicamide	Sigma	Cat# T9778
VU0357017 monohydrochloride	Sigma	Cat# V4640

Antibody name	Host	Source	Immunogen	Dilution<br><br>IHC/TEM	Secondary probe conjugated to gold nanoparticles in TEM
Ixori mAChR-A	R	This study	RTSSEDSTTLRTAEAGIDT-C	1:500/1:40	proteinA 10 nm (CMC Utrecht)
Ixori mAChR-B	R	This study	KRHKSKSENRARKALRTIS-C	1:500/1:40	proteinA 10 nm (CMC Utrecht)
Bommo myosuppressin (MS)	M	Yamanaka et al. 2006[4]	pEDVVHSFLRF-C	1:1000/1:40	Goat anti-mouse 10 nm (BBI)
Polpe FMRFamide	GP	Grimmelikhuijzen and Spencer 1984[8]	FMRFamide	1:1000/1:40	Goat anti-guinea pig 5 nm (BBI)
Bommo orcokinin	M	Yamanaka et al. 2011[9]	NFDEIDRS-C	1:1000/NA	NA
Drome SIFamide	R	Terhzaz et al. 2007[10]	AYRKPPFNGSIFamide	1:1000/NA	NA
Ixori FMRFa_MS-L	GP	This study	C-NARSNRIMHFamide	1:3000/NA	NA
Leuma leucokinin	R	Chen et al., 1993[11]	DASFHSWGamide	1:1000/NA	NA
Beta-3 Tubulin	M	Invitrogen (#MA1-118)	15 amino acid fragment of rat neuronal beta-3 tubulin	1:1000/NA	NA
Drome ChAT	M	Developmental Studies Hybridoma Bank University of Iowa	ChAT4B1, Mouse ChAT	1:200/NA	NA

Supplementary Table 2. List of primary antibodies used in this study.
Note that antibodies against *Bommo* MS, *Polpe* FMRFamide, *Bommo* orcokinin, *Dromme* SIFamide, and ChAT were validated for immunostaining in tick tissues in previous studies[3,5,12,13]. The underlined pE in the *Bommo* MS antibody indicates a pyroglutamate modification. NA, not applicable

REFERENCES

1. Dong, C. & Wu, G. Regulation of anterograde transport of alpha2-adrenergic receptors by the N termini at multiple intracellular compartments. *J Biol Chem* **281**, 38543–38554 (2006).
2. Maeda, S. *et al.* Structure and selectivity engineering of the M1 muscarinic receptor toxin complex. *Science* **369**, 161–167 (2020).
3. Mateos-Hernández, L. *et al.* Cholinergic axons regulate type I acini in salivary glands of Ixodes ricinus and Ixodes scapularis ticks. *Sci Rep* **10**, 16054 (2020).
4. Yamanaka, N. *et al.* Identification of a Novel Prothoracicostatic Hormone and Its Receptor in the Silkworm *Bombyx mori**. *Journal of Biological Chemistry* **280**, 14684–14690 (2005).
5. Šimo, L., Žitňan, D. & Park, Y. Two Novel Neuropeptides in Innervation of the Salivary Glands of the Black-Legged Tick, Ixodes scapularis: Myoinhibitory Peptide and SIFamide. *J Comp Neurol* **517**, 551–563 (2009).
6. Vancová, M. *et al.* Ultrastructural mapping of salivary gland innervation in the tick Ixodes ricinus. *Sci Rep* **9**, 6860 (2019).
7. Kaufman, W. R. Actions of some transmitters and their antagonists on salivary secretion in a tick. *American Journal of Physiology-Regulatory, Integrative and Comparative Physiology* **235**, R76–R81 (1978).
8. Grimmelikhuijzen, C. J. & Spencer, A. N. FMRFamide immunoreactivity in the nervous system of the medusa Polyorchis penicillatus. *J Comp Neurol* **230**, 361–371 (1984).
9. Yamanaka, N. *et al.* Bombyx Orcokinins Are Brain-Gut Peptides Involved in the Neuronal Regulation of Ecdysteroidogenesis. *J Comp Neurol* **519**, 238–246 (2011).
10. Terhzaz, S., Rosay, P., Goodwin, S. F. & Veenstra, J. A. The neuropeptide SIFamide modulates sexual behavior in Drosophila. *Biochem Biophys Res Commun* **352**, 305–310 (2007).
11. Chen, Y., Veenstra, J. A., Davis, N. T. & Hagedorn, H. H. A comparative study of leucokinin-immunoreactive neurons in insects. *Cell Tissue Res* **276**, 69–83 (1994).
12. Šimo, L., Slovák, M., Park, Y. & Zitnan, D. Identification of a complex peptidergic neuroendocrine network in the hard tick, Rhipicephalus appendiculatus. *Cell Tissue Res* **335**, 639–655 (2009).
13. Roller, L. *et al.* Orcokinin-like immunoreactivity in central neurons innervating the salivary glands and hindgut of ixodid ticks. *Cell Tissue Res.* **360**, 209–222 (2015).
