# Supplementary material for: Two types of axonal muscarinic acetylcholine receptors mediate formation of saliva cocktail in the tick Ixodes ricinus
Source: Nat Commun. 2026 Jan 23;17:2867. doi: 10.1038/s41467-026-68654-3 (PMC13022164; doi:10.1038/s41467-026-68654-3)
Supplement: Supplementary file 2 — Description of Additional Supplementary Files [file 41467_2026_68654_MOESM2_ESM.pdf]

Description of Additional Supplementary Files

File name: Supplementary Dataset 1

Description: Protein sequences used for the phylogenetic analyses in Fig. 1a.

File name: Supplementary Dataset 2

Description: List of saliva proteins from eight different experimental conditions in Fig. 7m, 7n and Supplementary Fig. 12.

File name: Supplementary Movie 1

Description: 3D reconstruction of a confocal image illustrating the synganglion. Double labeling with anti-mAChR-A (green) and anti-MS (red) highlights PcLNS1–2 and PcMNS1–5 neurosecretory cells and their axons, respectively, located on the synganglion surface as well as within the internal lobes.
