# Supplementary material for: Two types of axonal muscarinic acetylcholine receptors mediate formation of saliva cocktail in the tick Ixodes ricinus
Source: Nat Commun. 2026 Jan 23;17:2867. doi: 10.1038/s41467-026-68654-3 (PMC13022164; doi:10.1038/s41467-026-68654-3)
Supplement: Supplementary file 6 — Reporting Summary [file 41467_2026_68654_MOESM6_ESM.pdf]

nature portfolio

Corresponding author(s): Ladislav Simo

Last updated by author(s): 06/11/2025

Reporting Summary

Nature Portfolio wishes to improve the reproducibility of the work that we publish. This form provides structure for consistency and transparency in reporting. For further information on Nature Portfolio policies, see our Editorial Policies and the Editorial Policy Checklist.

Statistics

For all statistical analyses, confirm that the following items are present in the figure legend, table legend, main text, or Methods section.

n/a	Confirmed	
☐	☒	The exact sample size (*n*) for each experimental group/condition, given as a discrete number and unit of measurement
☐	☒	A statement on whether measurements were taken from distinct samples or whether the same sample was measured repeatedly
☐	☒	The statistical test(s) used AND whether they are one- or two-sided<br>*Only common tests should be described solely by name; describe more complex techniques in the Methods section.*
☒	☐	A description of all covariates tested
☐	☒	A description of any assumptions or corrections, such as tests of normality and adjustment for multiple comparisons
☐	☒	A full description of the statistical parameters including central tendency (e.g. means) or other basic estimates (e.g. regression coefficient) AND variation (e.g. standard deviation) or associated estimates of uncertainty (e.g. confidence intervals)
☐	☒	For null hypothesis testing, the test statistic (e.g. *F*, *t*, *r*) with confidence intervals, effect sizes, degrees of freedom and *P* value noted<br>*Give P values as exact values whenever suitable.*
☒	☐	For Bayesian analysis, information on the choice of priors and Markov chain Monte Carlo settings
☒	☐	For hierarchical and complex designs, identification of the appropriate level for tests and full reporting of outcomes
☒	☐	Estimates of effect sizes (e.g. Cohen's *d*, Pearson's *r*), indicating how they were calculated

Our web collection on statistics for biologists contains articles on many of the points above.

Software and code

Policy information about availability of computer code

Data collection	Roboocyte 2 system; Fluostar Omega; Glomax Discover; GPU-accelerated workstation; Leica DMI8; Olympus BX53; JEOL 1400 TEM; JEOL 2100F TEM; Gatan K2K2 Summit direct electron detector controlled byby SerialEM; Matlab; LightCycler 480 SYBR Green I Master; UltiMate 3000 RSLCnano system; timsTOF Pro mass spectrometer, CaptiveSpray source
Data analysis	NCBI; MEGA 11; GraphPad Prism 9;9; Maestro; Protein Preparation Wizard; CHARMM36 force field; SwissParam; Desmond; Leica Application Suite X;X; Adobe Photoshop; Adobe Illustrator; Imodmop (IMOD package); Findbeads3d (IMOD package); MAPS software; Apreo SEM with VolumeScope; TrakEM279; Microscopy Image Browser; Amira; MaxQuant software (v1.6.14) with the Andromeda search, engine; Perseus software 1.6.14.0; ImageJ 1.54g; J;J; Jalview 1.8.3–1.2.12_JVL.

For manuscripts utilizing custom algorithms or software that are central to the research but not yet described in published literature, software must be made available to editors and reviewers. We strongly encourage code deposition in a community repository (e.g. GitHub). See the Nature Portfolio guidelines for submitting code & software for further information.

Data

Policy information about availability of data

All manuscripts must include a data availability statement. This statement should provide the following information, where applicable:

- Accession codes, unique identifiers, or web links for publicly available datasets
- A description of any restrictions on data availability
- For clinical datasets or third party data, please ensure that the statement adheres to our policy

All data generated or analysed in this study are included in the article and the supplementary information files. Source Data supporting the findings are provided with the manuscript. Mass spectrometry proteomics data have been deposited in the ProteomeXchange Consortium via the PRIDE84 repository under dataset identifier PXD055362 (https://proteomecentral.proteomexchange.org).

Research involving human participants, their data, or biological material

Policy information about studies with human participants or human data. See also policy information about sex, gender (identity/presentation), and sexual orientation and race, ethnicity and racism.

Reporting on sex and gender	n/a
Reporting on race, ethnicity, or other socially relevant groupings	n/a
Population characteristics	n/a
Recruitment	n/a
Ethics oversight	n/a

Note that full information on the approval of the study protocol must also be provided in the manuscript.

Field-specific reporting

Please select the one below that is the best fit for your research. If you are not sure, read the appropriate sections before making your selection.

- [x] Life sciences
- [ ] Behavioural & social sciences
- [ ] Ecological, evolutionary & environmental sciences

For a reference copy of the document with all sections, see nature.com/documents/nr-reporting-summary-flat.pdf

Life sciences study design

All studies must disclose on these points even when the disclosure is negative.

Sample size	Sample sizes (n or N) are reported in the figure legends. No statistical method was used to predetermine sample size; sample sizes were chosen based on standard practice and experimental feasibility.
Data exclusions	No data were excluded except in oocyte physiological recordings, where oocytes lacking mAChR-A or mAChR-B expression were omitted, and in saliva proteomic analyses, where proteins identified by a single peptide or with a score below 40 among three technical replicates were excluded.
Replication	All experiments subjected to statistical analysis were performed with three biological replicates unless otherwise specified. Drug screening for agonistic and antagonistic effects on receptors was conducted with two biological replicates. Protein concentration measurements in low-volume tick saliva samples were performed using pooled saliva collected from a minimum of seven individual ticks, with each measurement carried out in three technical replicates. All replication attempts were successful.
Randomization	No randomization was applied during experimental design or data collection, as no distinct study groups were involved.
Blinding	No blinding was applied during data collection or analysis, as no distinct study groups were involved.

Reporting for specific materials, systems and methods

We require information from authors about some types of materials, experimental systems and methods used in many studies. Here, indicate whether each material, system or method listed is relevant to your study. If you are not sure if a list item applies to your research, read the appropriate section before selecting a response.

Materials & experimental systems

n/a	Involved in the study
☐	☒ Antibodies
☐	☒ Eukaryotic cell lines
☒	☐ Palaeontology and archaeology
☐	☒ Animals and other organisms
☒	☐ Clinical data
☒	☐ Dual use research of concern
☒	☐ Plants

Methods

n/a	Involved in the study
☒	☐ ChIP-seq
☒	☐ Flow cytometry
☒	☐ MRI-based neuroimaging

Antibodies

Antibodies used

Primary Antibodies:
Ixori mAChR-A - this study 1:500/1:40 IHC/TEM
Ixori mAChR-B - this study 1:500/1:40 IHC/TEM
Bommo myosuppressin (MS) - Yamanaka et al. 2006 (DOI: 10.1074/jbc.M500308200) 1:1000/1:40 IHC/TEM
Polpe FMRFamide Grimmelikhuijzen and Spencer 1884 (DOI: 10.1002/cne.902300305) 1:500/1:40 IHC/TEM
Bommo orcokini Yamanaka et al. 20119 (DOI: 10.1002/cne.22517) 1:500 IHC
Drome SIFamide Terhzaz et al. 2007 (DOI: 10.1016/j.bbrc.2006.11.030) 1:1000 IHC
Ixori FMRFa_MS-L - this study 1:3000 IHC
Leuma leucokinin - Chen et al., 1993 (DOI: 10.1007/BF00354786) 1:1000 IHC
Beta-3 Tubulin - #MA1-118) Invitrogen 1:1000 IHC
Drome ChAT, (AB_528122) Developmental Studies Hybridoma Bank University of Iowa 1:200 IHC

Secondary Antibodies:
Goat anti-Rabbit IgG (H + L), Alexa Fluor 488, A-11008, Thermo Fisher Scientific
Goat anti-Rabbit IgG (H + L), Alexa Fluor 594, A-11012, Thermo Fisher Scientific
Goat anti-Mouse IgG (H + L), Alexa Fluor 594, A-11005, Thermo Fisher Scientific
Goat anti-Guinea Pig IgG (H + L), Alexa Fluor 488, A-11073, Thermo Fisher Scientific
Protein A–Gold 10 nm Cell Microscopy Core (CMC Utrecht, Netherlands)
Goat anti-Mouse IgG (H + L)–Gold 10 nm, EM.GAM10/2, (BBI Solutions, UK)
Goat anti-Guinea Pig IgG (H + L)–Gold, 5 nm, EM.GAG5/2, (BBI Solutions, UK)

Validation

Validation of antibodies included antibody pre-adsorption with the corresponding antigen or replacement of the primary antibody with pre-immune serum (Supplementary Figs. 4 and 5). These controls were applied for the Ixori mAChR-A and -B antibodies as well as for the Ixori FMRFa_MS-L and Leuma leucokinin antibodies. The specificity of the Ir-mAChR-A and Ir-mAChR-B antibodies was further verified by immunocytochemistry of CHO cells transfected with ir-machr-a or -b, compared to mock-transfected cells (Supplementary Fig. 4a, b).

Antibodies against Bommo myosuppressin (MS), Polpe FMRFamide, Bommo orcokinin, Dromme SIFamide, and ChAT were validated for immunostaining in tick tissues in previous studies (Mateos-Hernández, L. et al. 2020, doi: 10.1038/s41598-020-73077-1; Šimo, L., et al., 2009, doi: 10.1002/cne.22182; Šimo, L., et al., 2009, doi: 10.1007/s00441-008-0731-4; Roller et al., 2015, doi: 10.1007/s00441-015-2121-z)

All secondary antibodies and the anti-Beta 3-tubulin antibody were commercially available and validated by the manufacturers.

Eukaryotic cell lines

Policy information about cell lines and Sex and Gender in Research

Cell line source(s)	Chinese hamster ovary (CHO-K1; 85051005, Sigma) cells and human embryonic kidney (HEK, 85120602, Sigma) cells were used for transient expression of receptors and corresponding reporters. Xenopus laevis oocytes were obtained from TEFOR Paris-Saclay, France.
Authentication	n/a
Mycoplasma contamination	All cells were tested free of Mycoplasma contamination.
Commonly misidentified lines (See ICLAC register)	No commonly misidentified lines were used.

Animals and other research organisms

Policy information about studies involving animals; ARRIVE guidelines recommended for reporting animal research, and Sex and Gender in Research

Laboratory animals	New Zealand rabbits, 11-12 weeks old, Charles River

	Oncins France 1 mice, 5-7 weeks ol, Charles River
Wild animals	NoNo wild animals were used.
Reporting on sex	Both the rabbits and mice were females, but sex was not considered iin this study.
Field-collected samples	NoNo field-collected samples were used.
Ethics oversight	All the experimental procedures were approved byby the local ethical committees: Animals use iin France was approved byby the ComEth Anses/ENVA/UPEC Ethics Committee for Animal Experimentation, (permit No. APAFIS #35511–2022022111197802 v2). IIn the Czech Republic, procedures complied with the Animal Protection Law ooff the Czech Republic No. 246/1992 Sb. (regulation 419/2012) and ethics approval No. 13/2021-P.

Note that full information on the approval of the study protocol must also be provided in the manuscript.

Plants

Seed stocks	n/a
Novel plant genotypes	n/a
Authentication	n/a
